# P19 Our experience diagnosing and treating deficiency of adenine deaminase 2 (DADA2) vasculitis

**DOI:** 10.1093/rap/rkad070.040

**Published:** 2023-09-27

**Authors:** Nawal Al Daqqaq, Ben Rhodes, Lorraine Harper

**Affiliations:** Queen Elizabeth Hospital, University Hospitals Birmingham, Birmingham, United Kingdom; Queen Elizabeth Hospital, University Hospitals Birmingham, Birmingham, United Kingdom; Queen Elizabeth Hospital, University Hospitals Birmingham, Birmingham, United Kingdom

## Abstract

**Introduction:**

Deficiency of adenine deaminase 2 (DADA2) is caused by biallelic pathogenic variants in the gene on chromosome 22q11. It is autosomal recessive with incomplete penetrance, and a variable presentation. It typically manifests as a systemic vasculopathy with early onset strokes, cutaneous manifestations, immunodeficiency and cytopenia. Traditional immunosuppressants have limited efficacy, but vasculitic manifestations have been reported to respond to biologic TNF-inhibitors. We report a patient with confirmed ADA2 deficiency whose cutaneous manifestations were previously classified as Sneddon syndrome. After a period of stability on adalimumab she developed marked cutaneous ulceration that healed following a therapy switch to intravenous infliximab.

**Case description:**

We report a 39-year-old female with a long clinical history that started in childhood when she developed Diamond-Blackfan anaemia that went into remission following corticosteroid treatment and transfusions. In 2006, she suddenly had two thalamic infarcts and was thoroughly investigated but was found to have unremarkable vascular imaging, a negative antiphospholipid syndrome and autoantibody testing, a modestly elevated CRP (5-15 mg/L) and a notably persistent low serum IgM. She continued to experience frequent migraines associated with transient sensorimotor symptoms. Subsequently, she developed recurrent botchy, polymorphic macular eruptions on the lower limbs. A deep cutaneous biopsy demonstrated lymphocytic predominant perivascular inflammation of deep dermal vessels associated with thrombus formation and endothelial proliferation. A putative diagnosis of Sneddon’s syndrome was made. However, after the subsequent identification of ADA2-deficiency vasculitis, the patient underwent genetic screening which identified a compound heterozygosity for two novel ADA2 mutations (c6634_636, delTTC (p.Phe212del) and c.1225C>T (p.Pro409Ser)). Her mother was as a carrier.

She was started on adalimumab injections to which she had a good clinical response with improved cutaneous disease and no further neurological symptoms. However, after two years of disease stability, she then developed a significant ‘punched out’ ulcer on the mid-calf which was painful and increasing in size to a maximum of 2 x 3 cm. Dermatology colleagues were reluctant to re-biopsy due to a high likelihood of non-specific appearances and increased risk of poor healing leading to worsening ulceration. Drawing on experience from rheumatoid arthritis that a switch in anti-TNF biological will often restore efficacy following secondary non-response to adalimumab; we empirically switched treatment to intravenous infliximab, with a reducing course of prednisolone (40mg per day tapering to 5mg after 12 weeks). This was associated with a notable improvement within one month and a complete healing of the ulcer within 4 months.

**Discussion:**

DADA2 remains a rare condition with a carrier frequency estimated at 1 in 236 persons worldwide and a predicted disease prevalence of approximately 1 in 222,000 persons. Patients with DADA2 who have haematological manifestations tend to present during early infancy, whereas delayed presentation is common in patients with vasculitis and systemic inflammation. It should be suspected in patients with early onset strokes, livedo and other haematological and immunological features. Additionally, many previously diagnosed cases of Sneddon syndrome may in fact have DADA2, with a low IgM level favouring the later. Diagnosis can be confirmed by genetic studies or by a biochemical assay that demonstrates near-absent levels of ADA2 activity in the plasma or serum. Interestingly, on screening of adult patients with polyarteritis nodosa (PAN) 3.4% possessed biallelic pathogenic ADA2 variants and similarly, in genomic survey of patients with Diamond-Blackfan anaemia 2% possessed biallelic pathogenic ADA2 variants in the absence of the ribosomal protein gene mutations associated with it. Treatment is phenotype based with a preference for lifelong TNF inhibitor and biosimilar use in those with vasculitis and/or systemic inflammation, though not currently commissioned for this indication. Glucocorticoids, disease-modifying antirheumatic drugs, and other biologic agents may partially ameliorate systemic inflammation. Unfortunately, they are all ineffective for severe hematologic manifestations and hematopoietic cell transplantation (HCT) is currently the only treatment option. Treating asymptomatic patients and mildly symptomatic patients picked up on screening remains senior clinician led on a risk benefit basis. Disease activity is clinically monitored with an overall mortality rate estimated at 8%.

**Key learning points:**

The main learning points for me included an increased awareness of this syndrome, its variable presentation, association with other autoimmune illnesses, diagnosis, and treatment initiation. In addition, I learnt about screening of family members, weighing the benefits versus risks when considering treating mildly symptomatic and asymptomatic patients, clinical monitoring for response, and when to switch to other anti-TNFs and biosimilars.

From this conference, I would like to explore the experience of our rheumatology colleagues with similar cases.

